# Recent Advances in the Determination of Veterinary Drug Residues in Food

**DOI:** 10.3390/foods12183422

**Published:** 2023-09-14

**Authors:** Rimadani Pratiwi, Shinta Permata Ramadhanti, Asyifa Amatulloh, Sandra Megantara, Laila Subra

**Affiliations:** 1 Department of Pharmaceutical Analysis and Medicinal Chemistry, Faculty of Pharmacy, Universitas Padjadjaran, Bandung 45363, Indonesia; shinta20003@mail.unpad.ac.id (S.P.R.); asyifa20001@mail.unpad.ac.id (A.A.); s.megantara@unpad.ac.id (S.M.); 2 Faculty of Bioeconomic, Food and Health Sciences, University of Geomatika Malaysia, Kuala Lumpur 54200, Malaysia; laila@geomatika.edu.my

**Keywords:** drug residue, food, veterinary drug, analytical method, analysis, maximum residue limits

## Abstract

The presence of drug residues in food products has become a growing concern because of the adverse health risks and regulatory implications. Drug residues in food refer to the presence of pharmaceutical compounds or their metabolites in products such as meat, fish, eggs, poultry and ready-to-eat foods, which are intended for human consumption. These residues can come from the use of drugs in the field of veterinary medicine, such as antibiotics, antiparasitic agents, growth promoters and other veterinary drugs given to livestock and aquaculture with the aim of providing them as prophylaxis, therapy and for promoting growth. Various analytical techniques are used for this purpose to control the maximum residue limit. Compliance with the maximum residue limit is very important for food manufacturers according to the Food and Drug Administration (FDA) or European Union (EU) regulations. Effective monitoring and control of drug residues in food requires continuous advances in analytical techniques. Few studies have been reviewed on sample extraction and preparation techniques as well as challenges and future directions for the determination of veterinary drug residues in food. This current review focuses on the overview of regulations, classifications and types of food, as well as the latest analytical methods that have been used in recent years (2020–2023) for the determination of drug residues in food so that appropriate methods and accurate results can be used. The results show that chromatography is still a widely used technique for the determination of drug residue in food. Other approaches have been developed including immunoassay, biosensors, electrophoresis and molecular-based methods. This review provides a new development method that has been used to control veterinary drug residue limit in food.

## 1. Introduction

The presence of drug residues in food has been a serious problem since they can cause harmful effects to humans. Drug residue in food generally comes from veterinary drugs that are widely used in animal husbandry. The use of veterinary drugs in food-producing animals includes preventing and overcoming animal diseases, increasing animal growth and improving the conversion rate of feed. These drugs can leave residue in their original compound form or their metabolites in food products [1]. Some of these residues can be accumulated and biomagnified through the food chain, which could be detrimental to human health if they exceed specific thresholds. According to the Food and Drug Administration (FDA), residue means any compound present in edible tissues that results from the use of a drug and includes the drug, its metabolites and any other substance formed in or on food because of the drug’s use. In the USA, regulatory tolerances for registered veterinary drugs are established by FDA in Title 21 Part 556 Tolerances for Residues of New Animal Drugs in Food [2]. In the European Union (EU), maximum residue limits of pharmacologically active substances in foodstuffs of animal origin are also strictly regulated through Commission Regulation (EU) No. 37/2010 [3].

Governments and associated agencies have established regulations, standards and procedures to monitor veterinary drug residues in the edible tissues of animals raised for food production. The objective is to ensure human health protection through the supervision and control of these residues [4]. For instance, the rising anxiety arises from the increasing occurrences of allergies and the development of antimicrobial-resistant microorganisms caused by the excessive utilization of veterinary antibiotics. Certain drugs, such as diethylstilbestrol, nitrofurans and chloramphenicol, have been prohibited due to their proven cancer-causing properties [5]. Considering the growing public attention and concern regarding food safety, coupled with the significant role of the meat industry in the global economy, the need for uncomplicated, automated and efficient analytical methods to monitor drug residues in food is expected to keep rising [4]. Current separation and detection methods are most often based on chromatography, immune affinity or biosensor [6,7].

Some reviews about veterinary drug residues in food have been published in previous years. Shuling Li et al. in 2020 [1] and Bo Wang et al. in 2021 [8] reviewed analysis of veterinary drug residues in food, including sample preparation methods and analytical techniques that were developed until 2020. These reviews reported various sample preparation methods such as solid-phase extraction, liquid–liquid extraction, immunoaffinity chromatography, accelerated solvent extraction, matrix solid-phase dispersion extraction and QuEChERS (quick, easy, cheap, effective, rugged and safe). Meanwhile, various analytical methods have been utilized for the detection of drug residues in food including microbial inhibition, enzyme-linked immunosorbent assay (ELISA), colloidal gold immunoassay, fluorescence polarization immunoassay, biosensor technology, application of quantum dots, capillary electrophoresis, liquid chromatography and liquid chromatography-mass spectrometry [1,8].

The current review will discuss a complete overview from describing the regulation of drug residue in food, classification of the drugs, types of food that we commonly find drug residues in it and provide an overview of the recently available analytical technique used for the detection of drug residue in samples from 2020 to the present. The results show that the analytical method has been developed; we found that chromatographic techniques are widely used for determining drug residue levels in food such as HPLC-UV (high-performance liquid chromatography-ultra violet), LC-MS/MS (liquid chromatography tandem mass spectrometry), GC–MS (gas chromatography–mass spectrometry), GC-MS/MS (gas chromatography tandem mass spectrometry), and MLC (micellar liquid chromatography). Another approach for detecting drug residues is through biosensors, for instance, lateral flow immunochromatographic biosensor assays and immunochromatographic strip biosensor, which involve the combination of biological components with chemical detectors to rapidly monitor the presence of drugs in animal derivative food. The use of immunoassay techniques like indirect competitive-ELISA, fluorescence polarization immunoassay, immunochromatography assay and electrophoresis techniques for the determination of drug residue was reviewed in this article. Other techniques are also reviewed in this article, including electrophoresis and molecularly imprinted polymers. 

## 2. Regulatory Overview of Drug Residues in Food

FDA and EU interpret drug residue as pharmacologically active substances (whether active principles, recipients or degradation products) and their metabolites which remain in foodstuffs obtained from animals to which the veterinary medicines in question have been administered [9]. After treating a food-producing animal with a drug for a certain period, traces of the drug might be present in or on food products made from that animal. This problem happens because of the administration of drugs to animals. Administering drugs to animals for the purpose of treating, preventing or controlling a disease or infection may affect their health and well-being. Moreover, when antibiotics are administered to animals intended for food production, it can lead to the presence of antibiotic residues in animal-derived products. The presence of drug residue in food products, such as meat, chicken, poultry, fish, ready meal or honey can pose substantial health risks to human consumers [10,11]. The presence of these residues can be caused by various factors, including failure to adhere to the recommended withdrawal period for each medication, use of doses exceeding the approved guidelines for animals, contamination of animal feed due to treated animal waste or the unauthorized use of antibiotics [12].

The global contamination of food products encompasses various substances, including microorganisms and their nitrates, nitrites, hormones, pesticide residues, antibiotics, metabolites, mycotoxins, heavy metals, dioxins, polychlorinated biphenyls (PCBs), genetically modified organisms (GMOs), toxic pigments and melamine [13]. Residues of drugs can arise from the therapeutic or prophylactic use of medications in animals raised for food [14]. The main veterinary drugs that have the potential to contaminate food products include antimicrobial drugs, sedative drugs, nonsteroidal anti-inflammatory drugs (NSAIDs), anticoccidials, growth promoters and anti-helminthic [15].

Numerous antibacterial and anticoccidial drugs are authorized for use in poultry to combat enteric and respiratory diseases. The residues of these drugs in food animals can have various potential adverse effects, including carcinogenic, allergic, toxic, neurologic disorders and microbiological effects [16]. Drug hypersensitivity refers to an immune-mediated response in sensitive individuals, which can also be an adverse side effect of drug residues. Drug allergies, specifically IgE-mediated reactions, are a subset of drug hypersensitivity reactions. These allergies are characterized by an allergic response to macromolecules such as proteins, carbohydrates and lipids. Allergic reactions to drugs can manifest as anaphylaxis, serum sickness, skin reactions or delayed hypersensitivity. Drug hypersensitivity reactions are often more commonly associated with antibiotics, such as penicillin, among other medications [17].

To safeguard public health and minimize the potential exposure to drug residues from food products, it is essential to monitor and trace residues in animal-derived foods. Means of monitoring and tracing depend on the establishment of reliable determination methods, and the development and optimization of such methods should consider the edible tissues and the corresponding maximum residue limits (MRLs) [18]. MRLs have been established by the USFDA and European Union [19].

The FDA ensures that any drug residues that may be present in or on these products are not harmful to people. The agency does this by setting the drug’s tolerance level and withdrawal period as part of the approval process for a drug intended for use in food-producing animals. The FDA’s Center for Veterinary Medicine (CVM) is responsible for ensuring that drugs are safe and effective for use in animals and that food derived from animals is safe for human consumption [20].

In addition to guidance from the FDA, the European Commission has provided guidance on drug residue regulation contained in Commission Regulation (EU) 37/2010, which covers pharmacologically active substances in food products of animal origin [3]. EU used to see the maximum residue levels (MRLs) range, namely the permissible range of drug residues present in food. MRL is defined as the maximum allowed concentration of residue in a food product obtained from an animal that has not received veterinary medicine or that has been exposed to a biocidal product for use in animal husbandry [21]. The development of highly sensitive analytical methods is necessary for the determination of veterinary drug residues.

Regulations related to the marketing of animal drugs in the EU and the FDA of the United States have several differences in their approaches and requirements, as shown in Table 1 [22]. The primary distinction between the two entities is rooted in the establishment of regulations by various nations. Furthermore, variations emerge in the marketing strategies employed by the FDA and the EU due to unique stipulations. Specifically, the FDA’s strategy is contingent on the nature of the drug and its intended purpose, while the EU undertakes a thorough evaluation of the product’s efficacy, caliber and safety. Lastly, a slight disparity exists in the safety assessment carried out by these two entities. For instance, within the EU, specific prerequisites such as the execution of clinical and pre-clinical trials must be satisfied prior to the granting of marketing authorization.

## 3. Drug Residues in Food

**Figure 1:**
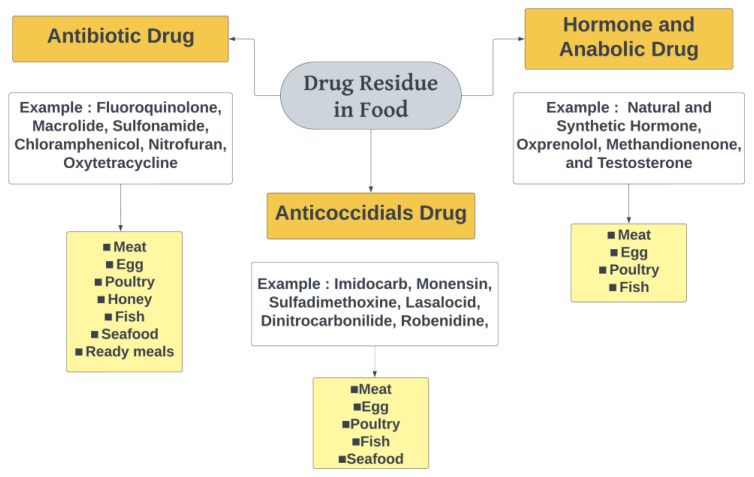
Drug residues in food.

Drug residues in food refer to the presence of remnants of drugs or their metabolites in edible products derived from animals or crops having been treated with pharmaceutical substances. When animals are administered drugs, such as antibiotics, anticoccidials, hormones or growth promoters, a portion of the drugs can be metabolized and excreted. However, small amounts of these drugs or their metabolites may remain in edible tissues, such as meat, milk, eggs, seafood or fish. A certain withdrawal period, which is the time required for the drug to be eliminated from the animal’s body, could determine the safety of the products [23]. The classification of drug residues in food is shown in Figure 1.

### 3.1. Classification of Drug Residues in Food

#### 3.1.1. Antibiotics

An antibiotic is a drug that can be used to treat or prevent human and animal diseases caused by bacterial infection. Furthermore, due to their notable medicinal benefits, the extensive utilization of antibiotics as feed additives becomes prevalent among animal foods. The presence of this drug residue in edible tissues results from the use of high concentrations of antibiotics in animals. The primary drug residues detected in the food were quinolones and sulfonamides, whereby ciprofloxacin, norfloxacin and sulfisoxazole emerged as the specific compounds exhibiting the most elevated concentrations [24]. The significant use of antimicrobial substances in food-producing animals plays a crucial part in producing antibiotic residues and contributes to the emergence of a global problem of antimicrobial resistance (AMR), allergic reactions, mutagenic effects, carcinogenic effects and even deaths [25].

#### 3.1.2. Anticoccidial Drugs

Anticoccidial drugs, also referred to as antiprotozoal agents, are used for the management of various microscopic parasitic infections, notably coccidiosis. Coccidiosis is a disease caused by protozoa belonging to the Eimeria genus. It primarily impacts poultry and ruminant animals [26]. Anticoccidials substances were administered as prophylaxis in poultry because the losses incurred due to coccidiosis were higher than the costs of providing preventive doses [27]. Some commonly found anticoccidial drugs in food include salinomycin, narasin, monensin, toltrazuril, robenidine, nicarbazin, dinitrocarbanilide, dimetridazole, decoquinate and lasalocid [26,28]. Anticoccidial residue in food exhibits undesirable toxicological effects, such as teratogenicity, hepatotoxicity or neurotoxicity, at high doses in laboratory animals [29].

#### 3.1.3. Hormones and Anabolic Drugs

Hormones and anabolic drugs are used for growth promotion, weight gain and increasing meat yield in livestock production [30,31]. The use of these drugs can result in residues of the active ingredients or their metabolites entering the human food chain [32]. Concerns regarding the increased use of these drugs have grown due todisturbances in the endocrine system’s function. Hormonal drugs are steroid or nonsteroidal products and synthetic substances that can mimic hormone functions [33]. In the European Union, the use of hormones and similar substances to enhance fattening, increase production and promote growth in livestock is prohibited, and the presence of residues is closely monitored [3]. Beta-agonists, such as clenbuterol, cimaterol, ractopamine, salbutamol and zilpaterol, are classified as growth promoters. Hormones can be classified into steroidal hormones and non-steroidal hormones (protein hormones). Steroidal hormones can be further divided into anabolic steroids and corticosteroids [34]. Hormones such as estrogen are known for their carcinogenicity and genotoxic potential, and others such as diethylstilbestrol are reported to have mutagenic, carcinogenic, immunotoxic and teratogenic properties [35].

### 3.2. Type of Food That Usually Contains Drug Residues

Residues of veterinary drugs are found in many animal-derived products such as meat, poultry, fish and ready meals. The use of hormones, growth promoters and antibiotics is indicated to ensure effective feeding and accelerated growth, and also as treatment and prophylaxis of animal diseases. However, inappropriate and excessive administration can cause harmful effects on humans, such as resistance, carcinogenicity, organ dysfunction and other adverse outcomes [36]. The drug residue detection in several food types is summarized in Table 2. 

#### 3.2.1. Meat

Meat samples are foods with a complex matrix in the presence of various compounds and various proteins and lipids can form suspensions [52]. At present, a lot of meat contains drug residues such as antibiotic, anticoccidial and human anabolite drugs through the provision of feed and water which then accumulates in the animal’s body and its products. Therefore, it is necessary to know the contaminants for consumer safety [53].

Direct analysis of meat samples will cause high matrix interference because meat has a complex matrix. Incompatible results are due to increased ionization so prevention is carried out by using solid phase extraction (SPE) as an effective sample preparation method. In addition, SPE can also increase sensitivity to a higher level. A study conducted by Temerdashev et al. in 2023 reported that the use of SPE with HLB cartridges in meat samples was suitable for the quantitative separation of methandionone, oxprenolol, and testosterone with high sensitivity, accuracy and precision [39].

#### 3.2.2. Egg

In general, eggs are a widely consumed food due to their complex matrix and high content of cholesterol, protein, phospholipids and other components. This could happen as a result of potential cross-contamination during food production, leading to the transfer of drug residues into the eggs [54]. To detect the presence of contaminants in eggs, a sensitive and reliable method is required. Egg samples necessitate pre-treatment to obtain an effective extraction solution. Based on the previous studies, SPE is commonly employed to extract egg samples, aiming to enhance the presence of target compounds. Like meat, SPE offers the advantage of amplifying target compounds through purification and extraction, resulting in increased sensitivity for detecting drug residues in eggs [41].

Studies conducted by Wang et al. in 2020 and Tang et al. in 2022 developed a combined method of SPE and liquid–liquid extraction (LLE) for the extraction and purification of egg samples [40,55]. The SPE-LLE method is considered to effectively enrich the extraction of target analytes in eggs. The researchers analyzed the tilmicosin residue with the GC–MS analysis method on poultry eggs. The results showed that a tilmicosin content of 18.9 μg/kg was found in one of the egg samples used [56].

#### 3.2.3. Poultry

According to the collected data, poultry ranks as the second most consumed meat worldwide. Poultry encompasses various animals, such as chickens, turkeys, quails, as well as waterfowl like ducks and geese [57]. Poultry farming is widespread in developing countries, which makes the availability of indiscriminate drug dosing easily accessible. The administration of drugs in poultry serves purposes such as prophylaxis, therapy and promoting growth. This includes the use of antibiotics and antiparasitic agents, which are commonly used in clinical medicine, leading to the accumulation of residues in the tissues that are consumed [58]. Residues can also originate from the contamination of poultry feed, the recycling of waste materials or the exposure of food substances to metals, toxic chemicals and similar compounds. These residues are responsible for the toxicity to humans [59].

Detection methods involving poultry samples were employed to determine the presence of enrofloxacin, sulfamethoxin and tylosine. The results showed that the concentration of these drugs exceeded the maximum residue limit (MRL) in the samples. Specifically, the amounts detected were enrofloxacin: 371 ± 139 μg/kg, sulfadimethoxine: 3750 ± 2180 μg/kg and tylosin: 4492 ± 1383 μg/kg, respectively. These findings highlight the necessity for action in drug administration to minimize excessive residues. The LC-MS method is widely employed for the detection of poultry samples, making it a suitable instrument for this purpose [60].

#### 3.2.4. Honey

Honey is the most preferred complex matrix of bee products due to its therapeutic abilities, nutritional value and delightful taste. Honey contains a high concentration of sugar and other components such as enzymes, proteins, vitamins and organic acids [61]. The specific content of honey can vary depending on environmental factors or season. Therefore, the sampling mechanism is a challenge to ensure that there are no contaminants, such as interfering matrices, present in honey. Inappropriate beekeeping practices such as giving antibiotics as a treatment can also cause residues in honey, which adversely affect human health [62]. From previous studies that have been carried out, honey detection using the biochip technology method is one of the antibiotic analyzers that can work simultaneously, quickly and cost-effectively [44,63]. The result of the analysis obtained is confirmed using the validation of the LC-MS/MS method. Meanwhile, for the preparation of honey samples, QuEChERS is usually used. Due to the complex nature of the honeycomb matrix, QuEChERS serves as an alternative for sample extraction as it has the capability to enhance sensitivity [64].

#### 3.2.5. Fish

Fish farming has experienced a significant increase in response to the rising market demand, making aquaculture a viable option for fish breeding. In aquaculture, it is common to utilize chemical compounds, including pharmaceuticals, in animal feeds to prevent and treat fish diseases. Therefore, it is important to detect chemical contaminants to analyze food safety to minimize human exposure to unwanted veterinary drugs [65,66]. For instance, the detection of robenidine hydrochloride in fish was conducted using the HPLC-HESI-MS/MS method, which was deemed simple, reproducible and accurate, yielding a concentration of 4.65 μg/kg with an additional concentration of 5 μg/kg. Various fish sample preparation methods are employed, and the QuEChERS method with simple modifications is widely utilized due to its advantages in terms of extraction efficiency, simplicity and short steps involved in obtaining the desired target samples [48].

#### 3.2.6. Seafood

Aquatic products continue to be a significant source of animal protein in the human diet, evident from the availability of 100 million metric tons of seafood each year. These aquatic products include various fish, shrimp, crabs and shellfish [66]. Therefore, it is important to pay attention to the safety and quality of aquatic products consumed, considering the presence of harmful residual drugs. Veterinary drugs are widely used to prevent and treat diseases in aquaculture farms that are susceptible to viruses, bacteria, parasites and fungi. These drugs are administered by incorporating them into the product feed [49]. However, it is essential to note that the dilution of drugs in water can pollute the water and soil environment, leading to exposure for aquatic animals. This exposure can occur through the trophic chain, resulting in public health problems such as increased risk of allergies, heightened carcinogen levels and antibiotic drug resistance [65]. 

The detection of drug residues in aquatic animals can be achieved using high-resolution mass spectrometry (HRMS) instruments, such as the hybrid quadrupole-Orbitrap. These instruments demonstrate the ability to simultaneously analyze many compounds, identify them and quantify the number of analytes in a sample. However, their effectiveness is occasionally restricted due to insufficiently accurate mass resolution [66].

In a recent study, the QuEChERS–LC-MS/MS method was developed to detect veterinary drugs in seafood. This method, which utilizes HPLC-MS/MS, has been established as a reliable approach for detecting trace amounts of antibiotic residues. Additionally, Lin et al. in 2021 developed a method that can be used for the detection of robenidine in shrimp and chicken which employs a monoclonal antibody-based indirect enzyme-linked immunosorbent assay (IC-ELISA) and a competitive immunochromatographic strip assay. The result of this study showed that the concentration of robenidine in shrimp was 10 µg/kg, which is below the maximum residue limit of 100 µg/kg for shrimp [50].

#### 3.2.7. Ready Meal

In addition to being found in raw food ingredients, drug residues can also be detected in ready-to-eat foods. This could be due to inappropriate cooking processes in the procedure, the type of drug used in animal production or cooking duration. Consequently, residues that were initially present in the raw tissue are not adequately destroyed. Salah and Ali reported that temperature had a significant effect on the amount of drug residue [67]. Their study results indicate that the heating process has effectively reduced drug residue levels in fast food, thus minimizing the toxic effects on humans [68]. 

In a study conducted by Frida et al. in 2016, to detect oxytetracycline (OTC) in ready-to-eat beef dishes in Tanzania using the LC-MS method, they observed that 25.7% of ready-to-eat beef samples contained OTC above the MRL of 200 μg/kg with the highest concentration recorded was 545.2 μg/kg. This poses a risk of microbial resistance in consumers due to the misuse of antibiotics during animal production. The LC-MS method is considered a fast, sensitive and easy means to detect residues and nonresidues in meat samples [51]. In addition, sample pre-treatment and extraction are carried out using the combined liquid–liquid extraction (LLE) approach because it is effective for the analysis complex samples [69].

## 4. Analysis of Drug Residues in Food

**Figure 2:**
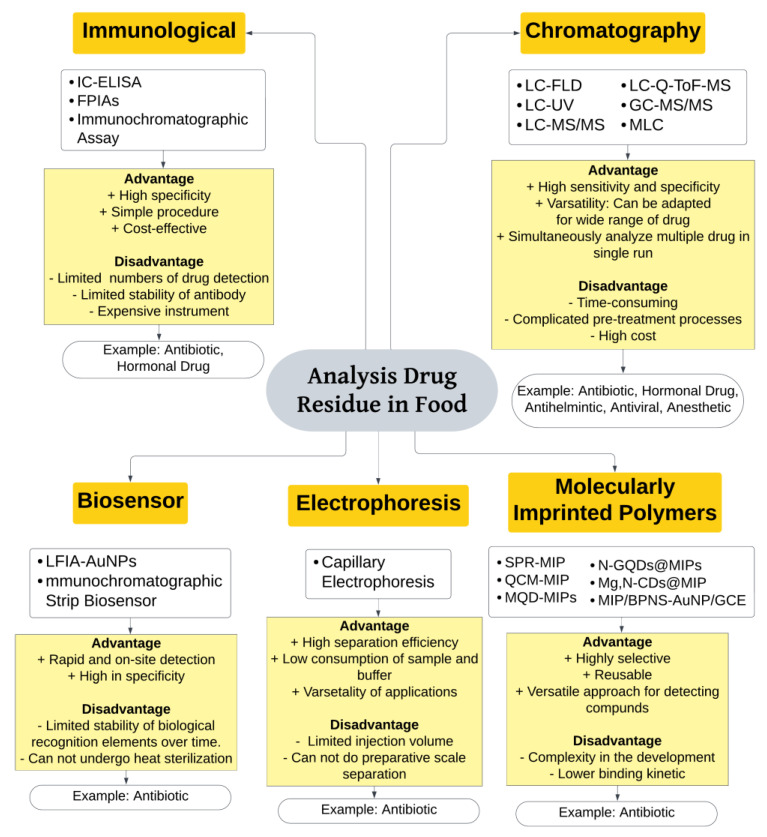
Analytical methods for the determination of veterinary drug residue in food, including their advantages and disadvantages: current update (2020–2023).

To ensure food safety, regulatory authorities establish maximum residue limits (MRLs) for various drugs. Monitoring and enforcement programs are implemented by regulatory agencies to ensure compliance with MRLs. Analytical techniques, such as immunological techniques, chromatography techniques such as liquid chromatography and gas chromatography, and biosensors are used to detect and quantify drug residues in food samples. Figure 2 presents a compilation of analytical techniques employed in the examination of drug residue in food, along with their respective advantages and disadvantages.

### 4.1. Immunological Technique

Immunoassays are antibody-based analytical methods for quantitative/qualitative analysis. The principle of immunoassays is based on specific antigen–antibody reaction. Table 3 provides a list of the application of immunological techniques to detect drug residues in food.

The first developed immunoassay was radioimmunoassay (RIA). It was used as a detector for endogenous plasma insulin. The radioisotope part was replaced by an enzyme for safety reasons which is now referred to as enzyme immunoassay/enzyme-linked immunosorbent assay (ELISA). ELISA is a technique for analyzing antigens with high sensitivity and selectivity. It has advantages such as a simple procedure, high specificity and efficiency, along with cost-effectiveness. However, there are disadvantages like the labor-intensive and costly antibody preparation process, the possibility of false results and antibody instability requiring chilled storage [74].

A current study about immunoassay for detecting veterinary drugs in food uses indirect competitive enzyme-linked immunosorbent assay (Ic-ELISA) to detect quinoxaline drug residues in food. Desoxquinoxalines (DQx) are quinoxaline desoxymetabolites that have been identified as the primary toxicant and emerging residue markers due to their high levels of presence. Ic-ELISA is a straightforward, convenient, sensitive, specific and cost-effective method used for analysis [70]. The difference between direct ELISA and indirect ELISA lies in the detection process. In direct ELISA, a primary detection antibody with a conjugated enzyme is added, which binds to the antigen that has coated the well. In indirect ELISA, after the immobilization of antigen, a primary antibody is added, which binds to the antigen and then followed by an addition of a secondary antibody with a conjugated enzyme and eventually, the substrate is added. Direct ELISA is a rapid diagnostic technique, but it cannot amplify signals, resulting in low sensitivity. The amplification step in indirect ELISA could enhance the sensitivity, so ic-ELISA can be more sensitive than direct ELISA [75]. As listed in Table 3, the limits of detection (LOD), limits of quantification (LOQ) and recoveries for the ic-ELISA assay of pork, swine liver, swine kidney, chicken and chicken liver were 0.48–0.58 μg/kg, 0.61–0.90 μg/kg, and 73.7–107.8%, respectively. These results indicate that ic-ELISA can effectively detect quinoxaline residue. The simplicity of this analytical method reduces the time required for sample pretreatment, improves efficiency and meets the demands of quinoxaline residue analysis. This is unlike instrumental detection assays such as HPLC or LC-MS/MS, which require costly equipment, complicated technology, a lengthy process and low detection efficiency [70].

Another method for detecting veterinary drug residues in food by immunological techniques is using fluorescence polarization immunoassays (FPIAs). FPIAs are widely utilized homogeneous techniques due to their advantageous features including sensitivity, reliability, rapidity and suitability for analyzing a large number of samples [76,77]. The fundamental principle of FPIA for small molecules involves the interaction between the tracer and competing antigens [1]. Some of these residues could be accumulated and biomagnified through the food chain and can be detrimental to human health if their levels exceed safe limits. As the concentration of analytes in the reaction solution increases, the analytes occupy the antibody binding sites, causing the tracer to bind less or not at all to the antibody, resulting in some free tracers. This can ultimately lead to a decrease in the FP value. FPIAs have gained significant attention and have been reported for detecting various small molecular compounds, such as veterinary drugs in food. Erythromycin in milk could be detected with FPIAs. The extraction of erythromycin from milk was done by precipitating the protein with the help of organic solvents. The LOD of erythromycin was 14.08 μg/L and the recovery was 96.08–107.77%. These findings demonstrated that FPIA exhibited satisfactory performance in terms of specificity, accuracy and precision when it comes to detecting erythromycin residues in milk [71]. FPIAs can also detect glucocorticoids in beef samples with LOD values reported as 0.27, 0.74, 3.19, 0.28, 1.56, 2.23 and 0.73 ng/mL for BMS, PNS, HCS, BCMS, CS, 6-α-MNPS and FHCS, respectively. Recoveries and RSD were obtained as 76.5–91.7% and 1.2–7.3%, respectively [72]. 

Immunochromatography assay or lateral flow tests are the underlying technology used in affordable, easy-to-use, fast and portable detection devices that are widely utilized in fields such as biomedicine, agriculture, food science and environmental sciences [78]. In addition, an immunochromatographic assay (ICA) was created using time-resolved fluorescent nanobeads (TRFNs) as a labelling technique for ultrasensitive detection of sulfamethazine in egg, honey and pork samples. The validity and accuracy of this assay were verified by comparing the results with those obtained from HPLC-MS/MS analysis [77].

### 4.2. Chromatographic Technique

Chromatography is a separation technique that involves the distribution of components between two phases: a mobile phase that carries the mixture through the system and a stationary phase that interacts with the components of the mixture. The separation is based on the principle of adsorption or partition [79]. Chromatography, particularly with various types of detectors, is commonly used to assess the concentration of veterinary drug residue levels in food. Table 4 presents a list of the chromatographic techniques that have been used to detect drug residues in food.

#### 4.2.1. Liquid Chromatography (LC)

Most commonly, separation and identification techniques rely on chromatography. These analyses typically employ chromatographic separation to aid in identification and can be conducted using liquid chromatography. The separation is based on the differential interactions of the sample components with the stationary phase and the mobile phase. As the sample travels through the column, different components interact with the stationary phase to varying degrees. Components that have stronger interactions with the stationary phase will move more slowly through the column, while those with weaker interactions will move more quickly [79]. Detection of the separated components is typically achieved using various detectors. Conventional detection methods such as ultraviolet (UV) or fluorescence detection (FLD) typically do not offer information on the structure of non-targeted compounds and face challenges when it comes to simultaneous detection of multiple substances [79]. Enrofloxacin and ciprofloxacin are second-generation fluoroquinolone, antimicrobial, that can be detected and determined by fluorescence detector [37]. Enrofloxacin and ciprofloxacin can emit fluorescence [93]. The analysis of enrofloxacin and ciprofloxacin by HPLC-FLD is presented in Table 4.

LC-UV can also be used for the detection of drug residues in food. For example, UV can be used to detect tetracyclines in food. Tetracycline has chromophore group that is capable of absorbing UV light, hence it can be detected by UV detector. The result shows that UV method can also have low detection limits (0.219–1.42 μg/kg) and quantification limits (0.731–4.72 μg/kg) and satisfactory relative recoveries (87.1–104%) [81].

Mass spectrometry (MS) detector has become the most powerful analytical tool for polar organic compounds determination at µg/kg, or even ng/kg level providing the sensitivity, selectivity and specificity needed to meet legislation [94]. There has been a shift toward employing ultra-high-performance liquid chromatography (UHPLC or UPLC) in combination with a mass analyzer. This switch to UHPLC has resulted in shorter analysis time while still allowing for the resolution of the same number of substances. Recently, analysis times have been extended to accommodate the increased number of substances being examined in a single run [95]. 

LC-MS/MS is the most widely used method for detecting drug residues in food. HPLC-MS/MS technology has gained significant interest due to its quick response time, excellent sensitivity, high accuracy, good separation capability, positive/negative ion mode compatibility and broad applicability in analyzing complex samples like swine, bovine, poultry eggs and chicken muscle, aquatic products, meat and milk [14,83,87,96,96]. The high sensitivity and specificity of MS detectors enable the application of generic sample preparation procedures, allowing for the simultaneous extraction of a wide range of residues with different properties. For instance, the ultraperformance liquid chromatography-tandem mass spectrometry (UPLC-MS/MS) was developed to simultaneously determine eight prohibited and restricted veterinary drugs and three metabolite residues across four categories including chloramphenicols, nitroimidazoles, lincosamides and macrolides with the validation data mentioned in Table 4 [89]. LC-triple-quadrupole tandem MS is a powerful technique that allows for the selective and sensitive quantification and confirmation of over a hundred target analytes in a single analysis. However, this method requires optimization of parameters specific to each compound and cannot be used for screening of non-targeted compounds [94]. The optimization of parameters specific to each compound was previously done by Hajrulai-Musliu et al. in 2021 using MS condition capillary voltage of 3.0 kV, source temperature of 150 °C, desolvation temperature of 400 °C, cone gas at 100 L/h and desolvation gas at 300 L/h. Three multiple reaction monitoring (MRM) transitions of banned analytes were chosen, while for analytes with permitted limits, two MRM transitions were chosen. The analysis of internal standards involved one MRM transition. Clenbuterol, amoxicillin and clenbuterol-d6 were the banned analyte, analyte with permitted limits and internal standard, respectively. The MRM parameter includes all + ESI: for ionisation mode, 276.97, 367.07 and 283.03 *m/z*; for precursor ion, 131.87, 167.77 and 202.95; 90.89, 159.94 and 203.56 *m/z* for product ion; 16, 30 and 30, and 16, 40 and 16 V of collision energy; 22, 28 and 22 V cone voltage [14].

Recently, Temerdashev et al. (2023) determined Oxprenolol, methandienone and testosterone in meat samples with UHPLC-Q-TOF-MS method [39]. Q-TOF-MS offers high sensitivity and mass accuracy for both precursor and product ions, enabling rapid elemental composition confirmation. As a gentle ionization technique, ESI has the ability to produce both molecules with added protons and molecules with removed protons. UHPLC provides high resolution for complex product separation and enhances the sensitivity of Q-TOF-MS [97].

The application of MLC, which is a subdivision of HPLC, has been shown to be a compelling option for the analysis of drugs in both solid and liquid forms found in food and biological waste [98]. In MLC, the mobile phases consist of hybrid micellar solutions composed of an anionic surfactant, commonly sodium dodecyl sulfate (SDS), along with a small amount of organic solvent. Micellar media possess a strong solubilization ability, enabling the dissolution of substances with varying degrees of hydrophobicity, including complex biological macromolecules like proteins. This is due to the presence of electrostatic, hydrophilic and hydrophobic sites within the micelles. The determination of albendazole and ivermectin in dairy products and biological waste from bovine and poultry was done using MLC [92]. After the optimization of chromatographic conditions, the system for testing was set with the optimum mobile phase an aqueous solution containing 0.15 M SDS, 6% 1-pentanol (*v*/*v*), pH 3, and the absorbance at 292 nm [92]. This method was successfully validated with validation data, as shown in Table 4 [92]. 

#### 4.2.2. Gas Chromatography

Besides using the liquid chromatography method, gas chromatography (GC) can also be used as a method to determine drug residue levels. Components in the mixture are distributed between two phases; one is a liquid stationary phase and the other is a mobile phase in the form of gas. GC is suitable for analyzing nonpolar and volatile compounds that are thermally stable. However, if the compounds are more polar or thermally unstable, an extra derivatisation step is necessary to make them compatible with GC [99]. 

Guo et al. (2021) designed GC-MS/MS method to analyze spectinomycin and lincomycin residues in eggs from various poultry species (e.g., chicken, duck, goose, pigeon and quail). In this study, an efficient pre-processing technique called accelerated solvent extraction (ASE) combined with solid-phase extraction (SPE) was employed for batch sample extraction and purification. The results demonstrated that the sample preparation process introduced fully automated technology to effectively minimize errors resulting from operator-related factors. The GC-MS/MS system exhibited a robust anti-interference capability, reducing matrix interference and providing higher sensitivity compared to alternative detection methods. The powerful anti-interference capability of the GC-MS/MS system can effectively reduce and provide lower sensitivity than other detection methods [91]. GC-MS/MS is regarded as a productive method for addressing the limitations of single-stage GC–MS. These limitations include the inability to adequately remove matrix interference, the occurrence of false positives and the incapability to simultaneously identify and quantifymultiple components.

Another study used GCMS/MS methods to detect tilmicosin in poultry eggs. Tilmicosin is an antibiotic that is commonly used in poultry farming. First, the extraction of the sample was done using LLE and SPE method. Then, the sample was derivatised with acetic anhydride to establish a confirmatory GC-MS/MS method to quantify the residual tilmicosin in poultry eggs. The results show that the linearity (R^2^) has a value around 0.9990. The limit of detection (LOD) for tilmicosin in chicken, goose and duck eggs was determined to be 3.8, 4.6, and 5.6 μg/kg, respectively. The limit of quantification (LOQ) for tilmicosin in chicken, goose and duck eggs was found to be 8.4, 9.6, and 10.5 μg/kg, respectively. The smaller the LOD and LOQ values, the greater its sensitivity [40].

### 4.3. Biosensor

Biosensors offer a convenient and cost-effective solution for quickly monitoring veterinary drugs by combining a biological component with a physicochemical detector. These sensors are highly appealing due to their simplicity and low cost. It canrapidly monitor veterinary drug residues [100]. The biological component, known as the bio receptor, can be an enzyme, antibody, nucleic acid or whole cell, and interacts with the analyte of interest. The physicochemical detector consists of two parts, namely transducer and electronics, and they convert the biological response into a measurable signal [101].

The development of drug residue detection in food using more sophisticated devices based on sensing principles, such as piezoelectric biosensors, optical biosensors, molecularly imprinted polymer biosensors, fluorescent biosensor or electrochemical biosensors, is an advanced method considered to work quickly, very sensitively and selectively. However, it has weaknesses, including not achieving an equivalent level of sensor detection limits and lower quantitative accuracy than traditional methods [102,103].

Among these, lateral flow immunochromatographic biosensor assays, which utilize biomolecules and nanoparticles, have emerged as an ideal and widely accepted analytical technology for on-site quantitative analysis of antibiotic residues [104]. Lateral flow immunoassay (LFIA) is a convenient and rapid paper-based technique used for detecting specific substances directly at the site of testing. The process involves adding the sample onto a self-contained device and obtaining the result within a few minutes [105]. This method relies on the interaction between an analyte or antigen (Ag) and a specific antibody (Ab) to form a complex known as the Ab–Ag complex. These assays are preferred due to their simplicity, speed, and cost-effectiveness [106]. Table 5 lists a summary of the electrophoresis technique used for detecting the drug residues in food.

A rapid and on-site detection method for enrofloxacin (ENR) in milk samples was developed by Alhammadi et al. in 2022 by utilizing a lateral flow immunochromatographic assay (LFIA) with the incorporation of gold nanoparticles (AuNPs). In this study, they developed LFIA test strips for the specific detection of enrofloxacin (ENR). They achieved this by combining the anti-enrofloxacin monoclonal antibody (ENR-Ab) with gold nanoparticles (AuNPs). Several optimization steps were implemented to enhance the sensitivity of the test strips. The results demonstrated a visual detection limit (vLOD) of 20 ng/mL, with a limit value of 50 ng/mL in milk samples. Importantly, the test strips exhibited minimal cross-reactivity with ENR analogues and other antibiotic components, highlighting their high specificity for ENR. The designed test strips were reliable, providing visual test results within 10 min and did not require any specialized equipment [107].

Another study was conducted by Lei et al. (2023) about determination of 83 antibiotic residues in aquaculture fish. A biosensor was designed to detect five different classes of antibiotic residues including 24 β-lactam antibiotics, 26 sulfonamides, five tetracyclines, 24 quinolones and four amphenicols. The objective of this research was to create a multiplex immunochromatographic strip biosensor (ISB) utilizing gold nanoparticles. The ISB multiplex comprised four components: a PVC backboard, a pad for absorption, a nitrocellulose (NC) membrane and a sample pad. A quantitative analysis was performed using the portable strip scanner to measure the color intensities of both the T-line and C-line. The T/C value refers to the ratio between the color intensity of the T-line and the C-line at a specific concentration of antibiotic. In aquaculture fish, detection process could be completed within 10 min. The results showed a recovery rate range between 87.5–115.2% and a coefficient of variation lower than 9.5%. The results obtained from this study showed ISB enables a simultaneous analysis of multiple classes of antibiotic residues in aquaculture fish using a single multiplex test system. It is a simple, cost-effective, reliable and high-performing method with significant potential for on-site analysis of antibiotic residues in aquaculture fish, providing rapid and relatively comprehensive results [108].

### 4.4. Electrophoresis

Electrophoresis is a technique that separates substances in a liquid by their electrical charge. It relies on the movement of charged particles in response to an electric field generated by two electrodes. The charged analytes migrate toward the electrode with the opposite charge, causing the constituents of a sample to be separated based on their ratio of charge to size. This process is commonly used in the separation of biopharmaceuticals such as peptides and proteins, and it can also serve as an alternative to liquid chromatography (LC) for small-molecule drugs. When electrophoretic separation is carried out using a gel, it is known as gel electrophoresis. This technique has been expanded to include various methods for separating proteins, such as sodium dodecyl sulfate polyacrylamide gel electrophoresis (SDS PAGE), isoelectric focusing (IEF) and Western blotting. Another approach is to perform electrophoretic separation in a capillary, known as capillary electrophoresis (CE). CE is a powerful method for quantifying substances [109]. Table 6 summarises the electrophoresis technique used for detecting drug residues in food.

Capillary electrophoresis (CE) has an important role in the development in the life sciences. In CE, the separation of analytes is based on their charge-to-size ratio when subjected to an electric field. This separation occurs within a silica capillary tube filled with a background electrolyte. In methods utilizing optical detection, the polyimide coating on the outer surface of the capillary is removed to create a detection window that allows light to pass through to the detector. The analytes are separated within the capillary by the influence of an electric field generated by a high-voltage power supply. During the process, a high-voltage power supply capable of delivering up to 30,000 V is utilized to apply the electric field across the capillary [113]. 

CE, as an electric field-mediated separation technique, was first introduced in 1981. It is now widely used to analyze molecules of different sizes in various applications, outperforming or complementing liquid chromatographic techniques [114,115]. CE is a cost-effective instrument that requires minimal reagents and offers high separation efficiency [116]. CE’s effectiveness can be enhanced through several methods including altering the buffer type, capillary type, pH, voltage and injection mode [117]. 

Based on a study conducted by Kośka et al. (2021), the optimization of electrophoretic condition was held by optimizing the concentration and pH of background electrolyte, and the amount of sample introduced into the capillary, voltage and temperature. The condition used for simultaneous determination of ciprofloxacin and ofloxacin in meat tissues included a phosphate-borate buffer with a concentration of 0.1 mol/L and pH 8.40 as the background analyte (consisting of 0.1 mol/L NaH2PO4 and 0.1 mol/L Na2B4O7). The sample was introduced into the capillary using hydrodynamic injection at a pressure of 60 mbar for 30 s. The analysis was conducted at a temperature of 25 °C, with a separation voltage of 16 kV. UV-Vis detection was performed at a wavelength of 288 nm for analytes [110]. The result of the validation data can be seen in Table 5. 

Nevertheless, the sensitivity of CE-UV detection is restricted as a result of the limited injection volume and narrow optical path. To overcome these limitations, online enrichment can be employed [111]. For determining the analytes, transient pseudo-isotachophoresis was applied to concentrate the sample directly on the capillary prior to the separation process. The technique of sample concentration using transient pseudo-isotachophoresis, which is also known as acetonitrile-salts stacking, was developed and introduced by Shihabi. This method is particularly useful for analyzing samples that already have a high concentration of salts. This technique allows for a larger sample injection volume, up to 30% of the capillary capacity, compared to the conventional capillary zone electrophoresis mode where the injection volume is typically limited to 1–3% of the capillary capacity [110]. To ensure the analyte remains within the desired band and prevent diffusion, the presence of a terminating ion with high field strength is crucial. In this regard, acetonitrile serves as a terminating pseudo-ion, providing the necessary high field strength to facilitate the migration of analytes without acting as an ion itself [111]. 

Another online enrichment technique was used by Yang et al. (2020) for the determination of sulfonamides in animal-derived products and water samples. The technique was called pressure-assisted electrokinetic injection (PAEKI). PAEKI is a technique that combines electrophoresis and electroosmotic flow (EOF), enabling ions to enter the capillary by the influence of an electric field, resulting in stacking. It offers a strong enrichment capability without compromising the efficiency of the separation process [111]. PAEKI could be used for anionic analytes, organic substances, inorganic substances and biological molecules [116]. The PAEKI-CE-UV method exhibited favorable enhancement factors (EFs), satisfactory linearity, high recovery and precision, and cost-effectiveness. It also enabled rapid and simultaneous determination of multiple analytes [111].

A different pre-concentration method, known as dynamic pH junction, was found to offer significant pre-concentration factors while maintaining the separation efficiency of amphoteric/zwitterionic compounds without the need for specific ampholytes [118,119]. The process of dynamic pH junction pre-concentration utilizes the difference mobility of zwitterionic analytes at various pH levels in both the sample solution and the background electrolyte (BGE) to accomplish concentration [120]. A study proposed a dynamic pH barrage junction pre-concentration method combined with CE-DAD (capillary electrophoresis with diode array detector) for quantifying six sulfonamide antibiotics (SAs) in milk and yogurt [112]. By injecting a sample matrix followed by a low pH acid zone, the sulfonamide antibiotics (SAs) were positively charged and migrated toward the cathode due to hydronium ions. Conversely, hydroxide ions from the high pH BGE caused negative charges and movement toward the anode. This concentration technique stacked the SAs at the sample-acid boundary, leading to narrower bands. Subsequent separation occurred in the basic BGE as in regular CE [112]. In the DAD (diode array detector), the detection cell receives polychromatic radiation, which is then dispersed by an optical grating after passing through the cell. The intensity of the dispersed radiation is captured by a series of photodiodes, with each diode recording radiation within a specific wavelength range. The signals from the photodiodes are rapidly recorded and stored [121].

### 4.5. Molecularly Imprinted Polymers (MIP)

Molecularly imprinted polymers also known as MIPs, can be described as synthetic counterparts of biological antibody-antigen systems. They function through a “lock and key” mechanism, specifically binding to the molecule they were templated on during their creation. The common fundamental process of MIP follows these steps: (1) during the production process, a polymer is created containing the template or target molecule attached to a functional group of the host either through covalent or noncovalent bonds; (2) the template molecule is removed from the polymer, resulting in a target-specific cavity ready for rebinding;(3) the MIP is exposed to the sample containing the target molecule, allowing the cavity to selectively uptake the target from a complex mixture [122]. MIPs have been widely used in sample pre-treatments, chromatographic separations, sensors, etc. MIPs can also be utilized as an analytical method for determining drug residues in food, as listed in Table 7.

The MIP method can be used to detect amoxicillin in eggs using surface plasmon resonance (SPR) and quartz crystal microbalance (QCM) sensors prepared through molecular imprinting technique. Polymeric films, composed of poly (hydroxyethyl methacrylate-methacrylic acid) (PHEMA-MAA), are created with amoxicillin-imprinted layers. These films served as the basis for SPR and QCM sensors. The amoxicillin-imprinted SPR and QCM sensors are employed to detect amoxicillin quickly, selectively and with high sensitivity in real samples. The SPR sensors possess a unique technology that allows the observation of biomolecular interactions in real time without the need for labeling. On the other hand, quartz crystal microbalance (QCM) sensors generate signals by detecting changes in the resonance frequency of piezoelectric crystals when they interact with the analyte. The LODs and LOQs for SPR and QCM are presented in Table 6. Because of the polymer’s ability to specifically bind to amoxicillin, both SPR and QCM sensors demonstrate a strong preference and selectivity for this antibiotic [123]. 

There are current studies about the detection of tetracycline in food using N-GQDs@MIPs and Mg,N-CDs@MIP methods [124,125]. N-GQDs@MIPs (nitrogen-doped graphene quantum dots coupled with molecularly imprinted polymers), a novel composite material, combines the benefits of molecular imprinting technology’s specificity with N-GQDs’ excellent fluorescent emission. It finds applications in target enrichment and sensing, attracting significant scientific interest [124]. Mg,N-CDs@MIP is another sensor-based fluorescent nano coupled with molecularly imprinted polymer. This method could also be used for a specific recognition and ultrasensitive detection of tetracycline in food. Mg,N-CDs exhibited excellent optical properties, making them a promising candidate for analysis applications. However, traditional CDs-based probes may face interference from coexisting substances due to their non-specificity. The developed Mg,N-CDs@MIP probe effectively addressed this issue by combining the exceptional optical performance of Mg,N-CDs with the superior recognition ability of MIP, resulting in a highly sensitive and selective detection of tetracycline molecules [125]. In comparing those two methods, Mg,N-CDs@MIP has lower LOD than N-GQDs@MIPs as shown in Table 6, resulting that Mg,N-CDs@MIP has higher sensitivity.

Another study about magnetic room-temperature phosphorescence quantum dots with molecularly imprinted polymers (MQD-MIPs) for a rapid detection of trace norfloxacin (NFX) residue in a complex food matrix was conducted by Chen et al. [126]. The highly selective probe was constructed by surface molecular imprinting technology using magnetic materials (Fe_3_O_4_ nanoparticles) as core, Mn-doped ZnS quantum dots (Mn-ZnS QDs) as phosphorescent materials and MIPs as selective recognition platform [126]. Another method was proposed to detect norfloxacin using MIP/BPNS-AuNP/GCE method. This study developed a robust MIP-based electrochemical sensor for detecting norfloxacin. The sensor was fabricated by electropolymerizing a polypyrrole-imprinted film onto a AuNP/BP nanosheet-modified glassy carbon electrode (BPNS-AuNP/GCE). The AuNP-decorated BP provided a large surface area for more specific imprinted sites, resulting in ultrasensitive NOR detection at nanomolar levels with excellent selectivity and stability. The sensor was successfully applied to detect NOR in pharmaceutical, food and environmental samples [127]. MIP/BPNS-AuNP/GCE offered higher sensitivity for detecting norfloxacin rather than MQD-MIPs.

## 5. Conclusions

The methods for analyzing veterinary drug residues in food have been summarized in this review. Some of these residues have the potential to accumulate and biomagnify through the food chain, posing risks to human health if they exceed certain levels. Governments and related agencies have established monitoring and regulatory laws, standards and procedures regarding veterinary drug residues in the edible tissues of food-producing animals to safeguard human health. Commonly found drug residues in food include antibiotics, hormonal and anabolic drugs, and anticoccidials. These foods, such as meat, eggs, honey, poultry, fish, seafood or ready meals have been detected to contain drug residues, especially antibiotic drugs.

There is a wide range of analytical methods available for detecting drug residues in food. Chromatographic techniques, particularly with various types of detectors, are widely utilized for determining drug residue levels in food. Another approach for detecting drug residues is through biosensors, which involve the combination of biological components with chemical detectors to rapidly monitor drug presence in animals. Biosensors offer a convenient solution due to their simplicity and low cost. However, they cannot undergo heat sterilization and have limited stability of biological recognition elements. The use of immunoassay techniques, based on specific antigen-antibody reactions, is another option. Immunoassays are valued for their simple procedures, high specificity and efficiency, although the process of preparing antibodies can be expensive and numbers of drug detection are limited. Electrophoresis methods allows the identification and quantification of drug residues in food samples offering advantages like high resolution and sensitivity, making it a valuable tool in determination of drug residues in food. Lastly, molecularly imprinted polymers (MIPs) offer an innovative method for detecting drug residues in food. Designed with specific binding sites for target drug molecules, these synthetic materials selectively recognize and bind to drug residues in food samples. 

The diverse range of analytical methods offers valuable tools for a reliable detection of drug residues in food. However, challenges and problems in veterinary drug residue detection still exist, such as a lack of screening methods and measurement of multi-drug residue detection in samples. Veterinary drug residue analyses that focus on finding a high-sensitivity method for screening and determining drug residue in samples are still being developed. 

## Figures and Tables

**Table 1:** Regulatory veterinary drug residue in FDA and EU.

	FDA	EU
Regulatory approach	The FDA regulates animal drugs through the U.S. Food, Drug and Cosmetic Act (FD & C Act) and the Federal Food, Drug and Cosmetic Act (FFDCA). The FDA applies separate requirements for different types of animal drugs.	The EU adopts an integrated approach known as the Centralized Licensing System. In this system, a single regulatory authority, the European Medicines Agency (EMA), is responsible for evaluating and granting marketing authorization for animal drugs throughout the EU member states.
Requirement for marketing	Based on category types of veterinary drugs such as prescription drugs, over-the-counter drugs and feed additive drugs, and their intended use.	Based on a comprehensive assessment of product effectiveness, quality and safety. In addition, evidence is needed regarding the benefits of using these drugs for animal health and public health.
Safety and effectiveness assessment	Requires strong evidence regarding the safety and effectiveness of veterinary drugs prior to granting a marketing authorization. Monitoring and reporting of adverse events is an important aspect of U.S. veterinary drug regulation.	Requires strict requirements regarding safety and effectiveness assessment of veterinary drugs by undergoing clinical and non-clinical testing prior to granting a distribution permit. Monitoring and reporting of adverse events is also an important part of EU veterinary drug regulation.

**Table 2:** Drug residues in several food type.

Food Type	Analyte	Method	Concentration (μg/kg)	Maximum Residue Limit (MRL) (μg/kg)	Reference
Meat	Enrofloxacin and Ciprofloxacin	HPLC-FLD	Enrofloxacin: 130.3–578.6 Ciprofloxacin: 15.7–28.8	100	[37]
	Imidocarb	Immunochromatography Assay	50.61	50	[38]
	Oxprenolol, Methandienone, and Testosterone	UHPLC-Q-ToF-MS	Oxprenolol: 0.007 Methandienone: 0.03 Testosterone: 0.004	-	[39]
Egg	Tilmicosin	GC-MS/MS	18.9	75	[40]
	Monensin, Salinomycin, and Lasalocid	HPLC-MS/MS and UPLC-MS/MS	HPLC-MS/MS: 0.82–1.73 UPLC-MS/MS: 0.81–1.25	-	[41]
	Pregnenolone, Progesteron, Testosteron, Androstenedione, and Estrone	LC-MS/MS	19–116.03, 9–89.8, 0.04–0.5, 0.05–21, 1.83–9.3	-	[42]
Poultry	Enrofloxacin, Sulfadimethoxin, and Tylosin	HPLC-DAD	371 ± 139, 3750 ± 2180, 4492 ± 1383	Enrofloxacin: 100 Sulfadimethoxine: 100 Tylosin: 200	[37]
	Dinitrocarbanilide	LC-MS/MS	99	200	[43]
Honey	Chloromaphenicol	Immunochemical assay	<0.9	1	[44]
	Nitrofurans metabolite	LC-MS/MS	0.12–0.74	Nitrofurans: 1.00	[45]
Fish	Sulfacetamide and Sulfamethoxypyridazine	UPLC-MS/MS	4.31 ± 0.70 0.75 ± 0.15	100	[46]
	Natural and Synthetic hormones	GC–MS	0.030–1.9	0.1–10	[47]
	Robenidine Hydrochloride	HPLC-HESI-MS/MS	4.63	5	[48]
Seafood	CAP TAP, FF, FFA *	HPLC-MS/MS	0.834–1.81, 0.0615–107, 0.261–243, no detect	1000	[49]
	Robenidine	Ic-ELISA and Immunochromatographic strip assay	10	100	[50]
Ready meal	Oxytetracycline	LC-MS/MS	251.40	200	[51]

* chloramphenicol (CAP), thiamphenicol (TAP), florfenicol (FF) and florfenicol amine (FFA).

**Table 3:** Immunological technique for the determination of drug residues in food.

Analyte	Sample	Analytical Method	Sample Extraction	Linearity (Correlation Coefficient)	LOD (µg/kg)	LOQ (µg/kg)	Recovery (%)	RSD (%)	Reference
Quinoxaline	Pork,	Ic-ELISA and LC-MS/MS	-	-	0.48–0.58	0.61–0.90	73.7–107.8	-	[70]
	Swine liver,								
	Swine kidney, Chicken, and Chicken liver								
Robenidine	Shrimp and Chicken	Ic-ELISA and immunochromatographic strip assay	Centrifugation	0.99932	-	-	87.8–102.0	3.2–5.9	[50]
Erythromycin	Milk	FPIAs	Precipitation	-	14.08	-	96.08–107.77	-	[71]
DMS, BMS, PNS, HCS, BCMS, CS, 6-α-MNPS, and HFCS *	Beef	FPIAs	-	-	0.23, 0.36, 0.75, 3.57, 0.31, 1.59, 2.26, and 0.74		76.5–91.7	1.2–7.3	[72]
						-			


Sulfamethazine	Egg, Honey and Pork	Immunochromatographic assay (ICA)	Centrifugation	-	0.016, 0.049, 0.029	-	90.5–113.9 82.4–112.0 79.8–93.4	-	[73]
Imidocarb	Meat	Immunochromatographic assay	-	-	0.45	-	84.5–101.2	-	[38]

* dexamethasone (DMS), betamethasone (BMS), prednisolone (PNS), hydrocortisone (HCS), beclomethasone (BCMS), cortisone (CS), 6-α-methylprednisone (6-α-MPNS), fludrocortisone acetate (HFCS).

**Table 4:** Chromatographic techniques used for the determination of drug residues in food.

Analyte	Sample	Analytical Method	Sample Extraction	Linearity (Correlation Coefficient)	LOD (µg/kg)	LOQ (µg/kg)	Recovery (%)	RSD (%)	Reference
Levamisole, Mebendazole, 5-hydroxymebendazole, 2-amino-5-benzoylbenzimidazole	Poultry eggs (Hen, Duck, and Goose)	HPLC-MS/MS	Dispersive liquid–liquid microextraction (DLLME)	≥0.9990	0.04–0.30	0.12–0.80	86.77–96.94	2.06–4.22	[80]
Tetracycline	Eggs and Chicken	HPLC-UV	Dispersive liquid–liquid microextraction (DLLME)	0.9986–1.000	0.219–1.42	0.731–4.72	87.1–104	0.853–8.62	[81]
Veterinary drug	Beef	HPLC-MS/MS	Oasis PRiME HLB solid phase extraction	≥0.9990	-	2.0–5.0	60–103	<20	[82]
Chloramphenicol, Thiamphenicol, Florfenicol, and Florfenicolamine	Aquatic products	HPLC-MS/MS	-	>0.992	<0.01	0.02	84.0–105	0.769–13.7	[83]
Nevirapine, Famciclovir, Abidox, Acyclovir, Imiquimod, Memantine, Amantadine, Oseltamivir and Morpholinoguanidine	Meat, Egg and Milk	HPLC-MS/MS	PRiME HLB solid phase extraction	0.9991–0.9998	0.1–0.5	0.3–1.5	82.3- 95.7	3.2~5.9	[84]
Chloramphenicol, Thiamphenicol, Florfenicol, and Florfenicol amine	Aquatic product	HPLC-MS/MS	Solid phase extraction (SPE)	>0.992	0.01	0.02	84–105	0.769~13.7	[83]
Robenidine hydrochloride	Fish	HPLC-HESI-MS/MS	liquid–liquid extraction (LLE)	≥0.9985	<2.5	<5.0	85.8–108.2	12.4	[54]
Anesthetics and Sedatives	Flatfish, Eels and Shrimp	LC-ESI/MSMS	Acetonitrile (ACN) only (for flatfish and eel) and 0.1% ammonium acetate in ACN (for shrimp)	>0.98	0.2–2.0	0.5–5.0	64.7–112.5	1.0–8.6	[85]
β2-agonists (Clenbuterol, Ractopamine, Salbutamol, and Terbutaline)	Fermented ham	UHPLC-MS/MS	Solid phase extraction (SPE)	0.997–1.000	0.1	0.3	76.0–102.0	1.8–13.3	[86]
Stimulant drugs	Pork, Egg and Milk	UPLC-MS/MS	Hydrolyzed with β-glucuronidase/aryl sulfate esterase in pH 5. 2 ammonium acetatebuffer	>0.99	0.3–0.6	1.0–2.0	65.2–117.0	1.3–14.4	[87]
Sulfacetamide, Sulfamethoxypyridazine, Sulfapyridine, Sulfadoxine	Fish and Shrimp	UPLC-MS/MS	Solid-liquid extraction (SLE)	>0.99	Fish: 0.07–0.42 Shrimp: 0.13–0.48	Fish: 0.24–1.32 Shrimp: 0.42–1.62	75–105	<20	[46]
Tigecycline, Four tetracyclines and Their three 4-epimer derivatives	Chicken muscle	HPLC-MS/MS	Solid-phase extraction (SPE)	-	0.06–0.09	200	89–98	5.0 and 6.9	[88]
Chloramphenicols, Nitroimidazoles, Lincosamides, and Macrolides	Eggs, Liquid Milk, Chicken and Freshwater fish	UPLCMS/MS	Solid phase extraction (SPE)	>0.99	0.050–0.500	0.20–1.5	65.3–108.0	0.40–21	[89]
Anthelmintics (including Benzimidazoles, Macrocyclic Lactones, Salicylanilides, Substituted Phenols, Tetrahydropyrimidines, and Imidazothiazoles)	Chicken muscle, Pork, Beef, Milk and Egg	LC-MS/MS	Liquid–liquid extractions (LLE)	≥0.9752	0.02–5.50	0.06–10	61.2–118.4	≤19.9	[90]
Natural and Synthetic Hormones	Meat and Fish	GC–MS	Solid phase extraction (SPE)	≥0.996	0.4–15.0	-	90–105	≤7	[47]
Enrofloxacin Ciprofloxacin	Meat	HPLC-FLD	-	>0.998	0.5 2.2	1.6 7.5	62.0.0–63.3 58.6–60.9	11.2–12.5 4.9–6.9	[37]
Oxprenolol, Methandienone and Testosterone	Meat	UHPLC-Q-ToF-MS	Solid phase extraction (SPE)	0.998, 0.995, 0.996	0.25, 1.25, 0.50	0.50, 2.50, 1.25	89–96	-	[39]
Nitrofurans metabolite	Honey	LC-MS/MS	Magnetic Solid phase extraction (MSPE)	0.99	0.1–0.3	0.3–1.0	>85	<12	[45]
Spectinomycin and Lincomycin	Poultry (Chicken, Duck and Goose)	GCMS/MS	Accelerated solvent extraction (ASE) and and solid-phase extraction (SPE)	0.9992–0.9998	2.5–4.6	5.7–7.6	79.7–94.2	1.2–3.5	[91]
Tilmicosin	Chicken, Goose, Duck eggs	GC-MS/MS	Liquid–liquid extraction (LLE) dan Solid phase extraction (SPE)	0.9990	3.8, 4.6, 5.6	8.4, 9.6, 10.5	78.11, 72.80, 74.82	1.75, 1.62, 1.46	[40]
Albendazole and Ivermectin	Cattle and Poultry	MLC	Batch stirring-assisted solid-to-liquid extraction (BSASLE)	>0.999	10	25	86.3–105.6	<12.2	[92]

**Table 5:** Biosensor technique for the determination of drug residues in food.

Analyte	Sample	Analytical Method	Linearity (R^2^)	LOD (ng/mL)	LOQ	Recovery (%)	RSD (%)	Reference
Enrofloxacin	Milk	AuNPs	0.9266	20	-	-	-	[107]
Antibiotic drug	Aquaculture fish	ISB multiplex	0.9981	-	-	87.5–115.2	<9.5	[108]

**Table 6:** Electrophoresis technique for determination of drug residues in food.

Analyte	Sample	Analytical Method	Sample Extraction	Linearity (R^2^)	LOD (µg/kg)	LOQ (µg/kg)	Recovery (%)	RSD (%)	Reference
Ciprofloxacin Ofloxacin	Meat tissues (Chicken liver, Chicken kidneys, Duck liver, and Turkey liver)	CE-UV	Liquid–liquid extraction	0.9988 0.9987	0.0994 0.0361	0.27 0.11	85–115	<15	[110]
SMZ, SMR, SMM, SDZ, SMX, SFA *	Milk, Pork, and Egg	CE-UV	-	0.9931–0.9994	1.8–16.3 8.3–63.8 5.2–47.8	6.1–50.3, 25.3–182.6, 16.4–147.5	89–107, 96–113, 95–109	1.7–6.7, 2.2–7.3, 1.6–6.8	[111]
SMX, SMZ, SDZ, SMM, SDM, SAc *	Milk and Yoghurt	CE-DAD	Liquid–liquid extraction	≥0.9940	4.1–6.3	12.9–19.8	81.2–106.9	5.3–13.7	[112]

* sulfamethazine (SMZ), sulfamerazine (SMR), sulfamonomethoxine (SMM), sulfadiazine (SDZ), sulfamethoxazole (SMX) and sulfacetamide (SFA/SAc), sulfadimethozine (SDM).

**Table 7:** MIP techniques for determination of drug residues in food.

Analyte	Sample	Analytical Method	Linearity (R^2^)	LOD (µg/kg)	LOQ (µg/kg)	Recovery (%)	RSD (%)	Reference
Amoxicillin	Egg	SPR *	-	0.0005	0.0019	97.50–98.75	0.916	[123]
		QCM *	-	0.0023	0.0076	96.00–99.00	1.664	
Tetracycline	Milk, Honey, Egg, Chicken muscle	N-GQDs@MIPs *	0.9980, 0.9982, 0.9993, 0.9997	1.41, 1.26, 2.97, 2.46	-	92.6–111.2	4.6	[124]
	Milk, Egg, Pork	Mg,N-CDs@MIP *	0.9976	0.79	-	78.6–98.7	3.3–5.0	[125]
Norfloxacin	Spiked fish and Milk	MQD-MIPs *	0.9993	0.80	-	90.92–111.53	<7	[126]
	Milk	MIP/BPNS-AuNP/GCE *	0.988	0.000003832	-	99.36–105.2	2.35–5.80	[127]

* surface plasmon resonance (SPR), quarts crystal microbalance (QCM), nitrogen-doped graphene quantum dots coupled with molecularly imprinted polymers (N-GQDs@MIPs), f magnesium and nitrogen co-doped carbon dots with molecularly imprinted polymer (Mg,N-CDs@MIP), magnetic room-temperature phosphorescence quantum dots with molecularly imprinted polymers (MQD-MIPs), molecularly imprinted polymer based on Au nanoparticle-functionalized black phosphorus nanosheet nanocomposite modified glassy carbon electrode (MIP/BPNS-AuNP/GCE).

## Data Availability

Data are available within the article.
